# Professional Experience Moderates Behavioral Performance and Sensorimotor-Related Cortical Activity During Scoring Decision-Making in Aerobic Gymnastics Judges: An fNIRS Study

**DOI:** 10.3390/jintelligence14100245

**Published:** 2026-10-04

**Authors:** Lu Yin, Siyu Guo, Le Tao, Bin Shao

**Affiliations:** 1 School of Physical Education, Shanghai University of Sport, Shanghai 200438, China; 2311811010@sus.edu.cn; 2 School of Psychology, Shanghai University of Sport, Shanghai 200438, China; 2321974009@sus.edu.cn; 3 College of Physical Education and Health, East China Normal University, Shanghai 200241, China; ltao@tyxx.ecnu.edu.cn

**Keywords:** aerobic gymnastics judges, professional experience, scoring decision-making, functional near-infrared spectroscopy, sensorimotor cortex

## Abstract

Understanding how professional experience shapes cognitive and neural resource allocation across different task demands is important for elucidating expert decision-making. This study examines whether professional experience influences behavioral performance and sensorimotor-related cortical activity across different scoring dimensions. To this end, we compared aerobic gymnastics judges with different levels of professional experience during execution score and difficulty score decision-making. Forty-nine judges were included, comprising 23 expert judges with FIG international judging qualifications and 26 less-experienced judges with Chinese national Level 1 qualifications. Participants viewed aerobic gymnastics action videos and completed execution score and difficulty score tasks while cortical hemodynamic activity was recorded using functional near-infrared spectroscopy (fNIRS). Oxygenated hemoglobin (HbO) β values estimated using a general linear model were used as the index of task-related cortical activation. Expert judges showed higher scoring accuracy than less-experienced judges in both tasks, whereas no significant between-group differences were observed in reaction time. Under the current accuracy criteria, difficulty score decisions showed higher overall accuracy, whereas execution score decisions required longer reaction times. After channel-wise FDR correction, significant judge group × decision type interactions were observed in CH13 and CH19, primarily localized to the right precentral and postcentral gyri, respectively. Both channels exhibited crossover patterns during the video observation period: expert judges showed greater task-related activation under the execution score requirement, whereas less-experienced judges showed greater activation under the difficulty score requirement. Exploratory brain–behavior analyses further indicated a between-group difference in the association between CH19 activation and reaction time during the video observation period under the execution score condition. These findings suggest that experience-related differences in judging may involve task-dependent recruitment of sensorimotor resources during scoring-related action observation. Given the cross-sectional design and age differences between groups, these findings should be interpreted as experience-related associations rather than causal effects of long-term professional experience.

## 1. Introduction

In competitive sports, judging is not a simple perceptual response or numerical judgment, but rather a complex cognitive decision-making process performed under time pressure (Russell et al., 2019; Raab et al., 2021). Judges must identify key technical features from continuous and rapidly changing movement information, match observed performance with sport-specific rules and scoring criteria, and form scoring judgments within a limited time. This process involves multiple cognitive components, including action perception, attentional allocation, rule retrieval, information integration, and decision execution (Lee et al., 2019; Spitz et al., 2016; Flessas et al., 2015; Samuel et al., 2024). Compared with typical laboratory-based decision-making tasks, sports judging has higher ecological validity and provides an important research window for understanding the cognitive and neural mechanisms of complex decision-making in naturalistic contexts (Klein, 2008, 2015). Particularly in aerobic gymnastics, judges are required not only to continuously track the spatiotemporal dynamics of athletes’ movements, but also to simultaneously integrate multidimensional information, including movement structure, technical execution quality, and rule-based criteria, to make immediate scoring decisions (Flessas et al., 2015; Ste-Marie, 1999, 2000). Therefore, scoring by aerobic gymnastics judges is not only directly related to competitive fairness, but also provides an important research context for examining the cognitive processing mechanisms underlying complex action-based decision-making.

Previous studies have shown that professional experience significantly influences judges’ scoring performance and perceptual-cognitive processing. Compared with lower-level judges, high-level or expert judges demonstrate advantages in scoring accuracy, decision consistency, and the extraction of key movement-related information (Ste-Marie, 1999, 2000). This expert advantage may arise from specialized knowledge structures, representations of sport-specific rules, and action-pattern recognition abilities developed through long-term judging practice (Pizzera & Raab, 2012; Pizzera, 2015; Plessner & Haar, 2006). In other words, expert judges are not only able to identify critical movement information more accurately, but may also extract action features, match them with rule-based criteria, and make scoring decisions in a more efficient manner. However, existing research on judges’ professional experience has mainly focused on behavioral manifestations of expert advantage, such as scoring accuracy, visual search strategies, and perceptual cue extraction (Lee et al., 2019; Spitz et al., 2016; Moore et al., 2019; Wu et al., 2024). Insufficient evidence is available regarding how professional experience influences the neural processing mechanisms involved in scoring, particularly the neural implementation of different scoring tasks.

In technical judged sports, judges’ scoring is typically not based on a single-dimensional judgment, but rather consists of multiple scoring dimensions. Taking the scoring system of World Gymnastics, formerly the International Gymnastics Federation (FIG), as an example, judges are required to make separate judgments on the difficulty score and execution score. However, previous studies have often treated judges’ scoring as a unified evaluative process, with limited attention paid to potential cognitive differences between different scoring dimensions. In fact, difficulty and execution scoring may impose distinct information processing demands. Difficulty scoring primarily involves matching action categories, movement structures, and rule-based criteria. Because the information to be processed is relatively discrete and structured, difficulty score decision-making may be more closely related to rule-based category learning and action classification processes (Seger & Miller, 2010; Maddox et al., 2010; Milton & Pothos, 2011; Chase & Simon, 1973; Gobet, 1998). In contrast, execution scoring requires continuous evaluation of movement stability, fluency, control quality, and movement deviations. Its processing may therefore rely more heavily on the perception of overall movement quality, retrieval of action representations, and sensorimotor integration (Jeannerod, 2001; Rizzolatti & Craighero, 2004; Caspers et al., 2010; Grèzes & Decety, 2001). Thus, differences between scoring dimensions may not only reflect differences in scoring content, but may also correspond to distinct levels of perceptual-cognitive processing.

From a cognitive neuroscience perspective, the effects of professional experience on brain activity do not necessarily manifest as a unidirectional increase or decrease in activation. On the one hand, as professional knowledge structures and action representations become increasingly automatized, experts may perform familiar tasks with fewer neural resources, reflecting greater neural efficiency (Del Percio et al., 2008; Neubauer & Fink, 2009; Li & Smith, 2021). On the other hand, long-term training may also enhance the functional specialization of task-relevant neural systems, enabling experts to recruit neural resources selectively according to specific information processing demands (Neumann et al., 2016; Yang, 2015; Li & Smith, 2021). Thus, professional experience may be more likely to alter the mapping between task demands and sensorimotor processing than to simply increase or decrease overall brain activation. In judging tasks, difficulty score and execution score decisions require the extraction of different types of information; accordingly, their reliance on the sensorimotor system may also vary as a function of professional experience. Less-experienced judges, whose professional representations and rule knowledge are not yet highly organized, may rely more heavily on online action analysis and sensorimotor mapping. In contrast, expert judges may draw on more structured action representations and scoring knowledge to allocate neural resources more selectively according to different decision demands (Ste-Marie, 1999, 2000; Zentgraf et al., 2014). Therefore, the key question is not whether experts consistently show stronger or weaker activation, but whether professional experience alters the pattern of neural differences between execution score and difficulty score decisions.

Functional near-infrared spectroscopy (fNIRS) has been widely applied in research on sport cognition and action observation in recent years because of its relatively high ecological validity, good tolerance to movement, and suitability for dynamic task contexts (Herold et al., 2018; Yücel et al., 2021). It therefore provides an appropriate method for examining cortical hemodynamic changes during judging and scoring. fNIRS can record task-related cortical activity in relatively naturalistic action observation and video scoring contexts, and changes in oxyhemoglobin (HbO) concentration can be used to reflect the level of cortical activation (Herold et al., 2018; Yücel et al., 2021). During action scoring, judges need to match observed movement features with internalized scoring rules and action representations. This process may involve the representation, prediction, and integration of action-related information in sensorimotor-related cortical regions. However, existing studies have not directly compared the neural activity characteristics associated with execution score and difficulty score decisions under identical action-stimulus conditions. It also remains unclear whether professional experience modulates the extent to which different scoring dimensions rely on sensorimotor-related cortical regions.

In summary, this study combines behavioral measures with functional near-infrared spectroscopy (fNIRS) to examine behavioral performance and sensorimotor-related cortical activity during the video observation period under execution score and difficulty score requirements in aerobic gymnastics judges with different levels of professional experience. We propose the following expectations: first, expert judges would show higher scoring accuracy than less-experienced judges in both scoring tasks (Mann et al., 2007; Gobet, 1998); second, the two scoring tasks would differ in reaction time and in the current accuracy measure, although cross-task comparisons of accuracy should be interpreted in light of their different correctness criteria; and third, judges with different levels of professional experience would exhibit different patterns of sensorimotor-related cortical activity during the video observation period under the execution score and difficulty score requirements (Neubauer & Fink, 2009; Shiffrin & Schneider, 1977).

## 2. Methods

### 2.1. Participants

A total of 50 aerobic gymnastics judges were recruited for this study through the Chinese Aerobics Association. Of these, 24 judges holding FIG international judging qualifications were assigned to the expert group, whereas 26 judges holding Chinese national Level 1 judging qualifications were assigned to the less-experienced group. According to the International Gymnastics Federation (FIG) General Judges’ Rules 2025–2028, FIG international judges are classified into Brevet Categories 1–4. Among the 24 international judges recruited in this study, one was classified as Brevet Category 1, 10 as Brevet Category 3, and the remaining 13 as Brevet Category 4. It should be noted that the “Level 1 judges” in the less-experienced group refer to the Chinese domestic judging qualification system and are not equivalent to FIG Brevet Category 1. The Chinese domestic judging qualification system includes Level 2 judges, Level 1 judges, national-level judges, and international-level judges. Therefore, based on judging qualification level and officiating experience, judges with FIG international qualifications and extensive officiating experience were classified as experts, whereas Chinese national Level 1 judges were classified as less-experienced judges. The expert group had a mean age of 40.4 ± 4.6 years and a mean officiating experience of 14.5 ± 5.6 years, with 18 men and 5 women. One international-level judge (Brevet Category 4) did not complete the full experimental procedure and was therefore excluded, resulting in a final expert group sample of 23 participants for statistical analysis. The less-experienced group had a mean age of 26.4 ± 2.8 years and a mean officiating experience of 2.0 ± 1.7 years, with 21 men and 5 women.

The study was approved by the Research Ethics Committee of Shanghai University of Sport (protocol code 102772025RT162). According to a standard handedness inventory (Oldfield, 1971), all participants were right-handed. During the experiment, none of the judges reported a history of neurological disorders or the use of medications acting on the central nervous system. All participants provided written informed consent before taking part in the experiment.

The sample size was estimated using G*Power software (version 3.1.9.7; Faul et al., 2007). Assuming an effect size of f = 0.25, a significance level of α = 0.05, statistical power of 1 − β = 0.80, numerator degrees of freedom of 1, two groups, and two repeated measurements, the expected effect was considered to be of medium magnitude. The results indicated that a minimum total sample size of 34 participants was required to detect between-group differences. Considering a potential attrition rate of approximately 20%, a total of 50 aerobic gymnastics judges were ultimately recruited. After the experiment, one international-level judge (Brevet Category 4) was excluded because he did not complete the entire experimental procedure. Therefore, 49 participants were included in the final statistical analysis.

### 2.2. Scoring Task Design

The scoring decision task was assessed by having participants watch video clips of aerobic gymnastics difficulty elements. All stimuli were video clips obtained from the official judges’ learning platform of the World Gymnastics (WG) and were extracted from aerobic gymnastics World Championships and World Cup competitions held within the past two years. The videos were presented on an LCD monitor with a resolution of 1920 × 1080 pixels. E-Prime 3.0 software was used to record reaction time (RT) and accuracy (ACC) during the experiment.

To ensure the content validity of the experimental materials, three international-level judges, none of whom participated in the formal experiment, were invited to form an expert review panel and evaluate the suitability of the video materials on a 1–10 scale. The three judges rated the videos independently, and only video materials that received scores greater than 8 from all three judges were included (Shui et al., 2024). Subsequently, the expert review panel further evaluated the selected videos in terms of execution score and difficulty score. The results on which all three judges agreed were used as the standard answers, whereas materials with inconsistent ratings were excluded. To further evaluate the reliability of the expert criterion, inter-rater reliability was calculated using all 82 candidate video clips before consensus-based exclusion. Separate two-way random-effects, absolute-agreement, average-measures intraclass correlation coefficients [ICC (2,3)] were calculated for execution and difficulty scoring. Inter-rater reliability was excellent for both dimensions: ICC (2,3) = 0.995, 95% CI [0.993, 0.997], for execution scoring and ICC (2,3) = 0.997, 95% CI [0.996, 0.998], for difficulty scoring. Ultimately, 62 videos were selected, of which 2 were used as practice materials and 60 were used as formal experimental materials. The 60 formal experimental videos were balanced in terms of difficulty score and execution score.

During the experiment, the 60 formal videos were presented twice, once for execution score decisions and once for difficulty score decisions, resulting in a total of 120 trials. At the beginning of each trial, a cue indicating the decision type (execution score or difficulty score) was first presented on the screen. After participants pressed the spacebar to confirm that they understood the task requirement, a red fixation cross (“+”) was presented at the center of the screen for 1000 ms, followed by a 3000 ms video stimulus. After the video ended, the scoring interface appeared, and participants were required to enter a specific score ranging from 0 to 10 within 5000 ms. The permissible response set consisted of the integers 0–10, entered using the numeric keypad. Reaction time was measured from the onset of the scoring interface until completion of the full response entry rather than until the first keypress. RT analyses included only correct-response trials; trials with no response and trials exceeding ±3 SD from the participant’s condition-specific mean RT were excluded. Following the evaluation principles used in the FIG/World Gymnastics international judging examinations for aerobic gymnastics, in which difficulty judges and execution judges are assessed using different scoring criteria, this study adopted different accuracy criteria for the two tasks. For difficulty scoring, a response was considered correct only when it exactly matched the expert reference score. For execution scoring, a certain degree of deviation from the expert reference score was permitted, and responses within ±1 point of the expert reference score were classified as correct. It should be noted that the ±1-point criterion was an experimental operationalization based on the above evaluation principles rather than a direct adoption of the specific tolerance values used in FIG judging examinations (International Gymnastics Federation [FIG], 2024). Because the permissible response set consisted of 11 integer values (0–10), the nominal chance level under equiprobable random responding was 1/11 (9.09%) for difficulty scoring. For execution scoring, the nominal chance level was generally 3/11 (27.27%) when the reference score was not at a scale boundary because the reference score and values ±1 were accepted; at the boundaries (0 or 10), the corresponding nominal chance level was 2/11 (18.18%). These chance levels are descriptive only because actual response probabilities were not assumed to be uniform.

**Figure 1:**
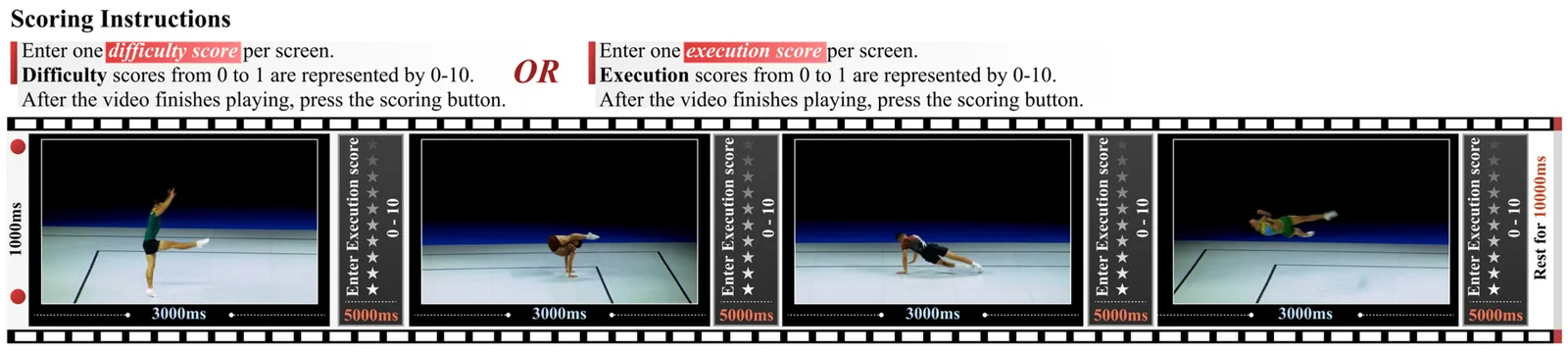
Flowchart of the scoring task. All video clips had corresponding standard answers for both execution scores and difficulty scores. The decision types were categorized as difficulty scoring and execution scoring.

The experiment adopted a block design (Catteeuw et al., 2009), in which the difficulty score and execution score tasks were presented in separate blocks, with each block containing only one task type. The order of the blocks was counterbalanced across participants. The experiment consisted of 30 test blocks, each comprising four trials, with a 10 s rest interval between blocks. The entire experiment lasted approximately 30 min. The video-based decision-making paradigm used in this experiment was adapted from previous studies on video-based decision-making tasks in sports officials (Kittel et al., 2019). The overall procedure of the scoring task is illustrated in Figure 1.

### 2.3. fNIRS Data Acquisition

**Figure 2:**
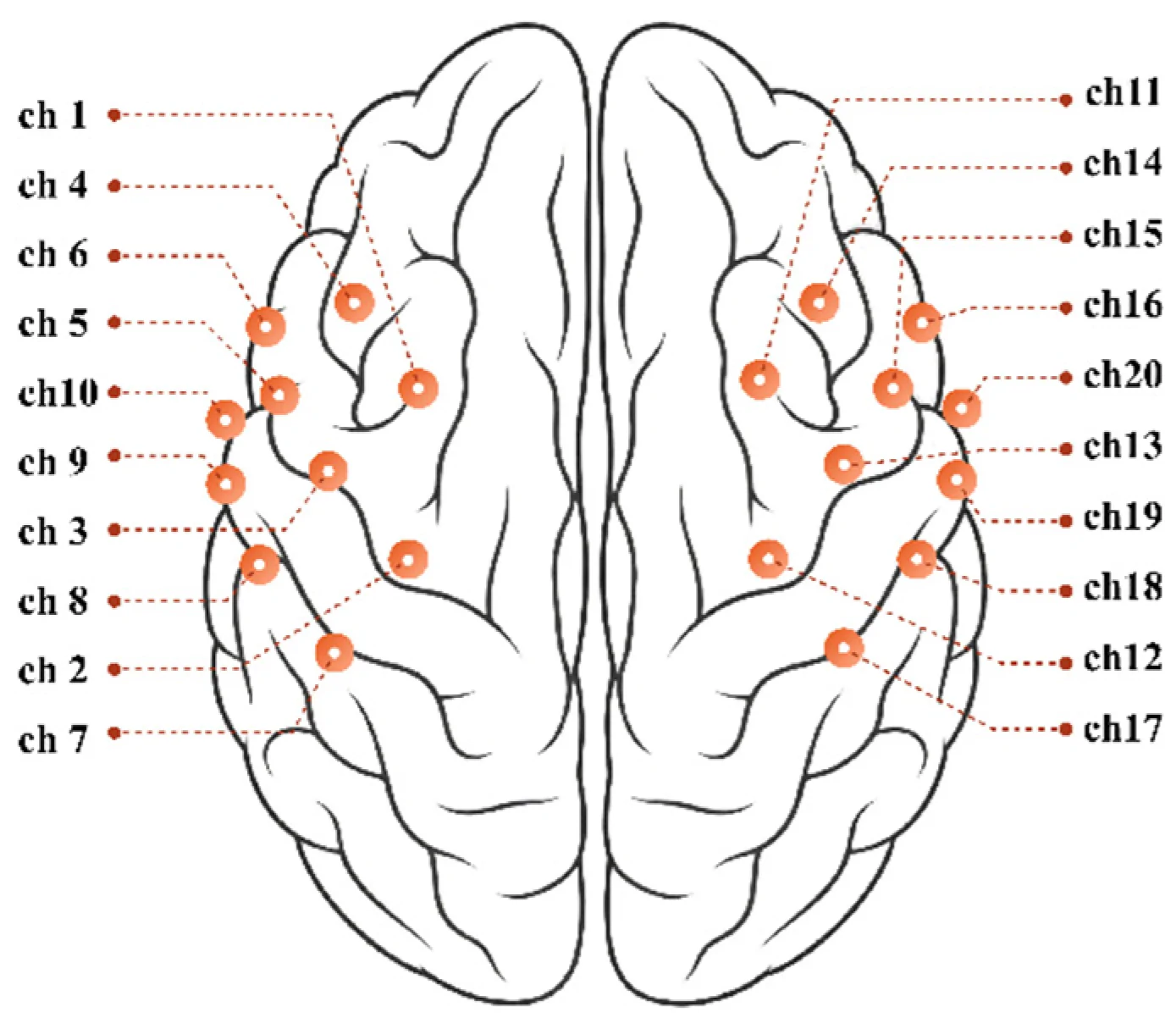
Schematic illustration of the fNIRS channel arrangement. CH1–CH20 indicate measurement channels 1–20.

Cerebral hemodynamic responses during the scoring task were recorded using a portable functional near-infrared spectroscopy system, NIRSport™ (NIRx Medical Technologies, Glen Head, NY, USA). The system consisted of 8 LED light sources and 7 photodiode detectors, with an interoptode distance of approximately 30 mm, forming a total of 20 measurement channels. These channels mainly covered the bilateral sensorimotor cortices and adjacent frontoparietal association regions. The sampling rate was 10.1725 Hz, and the emitted wavelengths were 756 nm and 853 nm. fNIRS caps of different sizes were selected according to each participant’s head circumference and were positioned in accordance with the international 10–20 EEG system. Spatial registration and anatomical labeling were then performed using Atlas Viewer and the AAL template (Figure 2). Based on this registration, the primary anatomical localization of each channel was determined according to its maximum overlap with regions defined by the AAL atlas, and channels sharing the same primary anatomical localization were grouped into the corresponding regions of interest (ROIs). The ROI classification, primary anatomical localization, and overlap percentage for each channel are presented in Table 1. Because individual structural MRI and 3D digitization were not available, these anatomical labels should be interpreted as atlas-based probabilistic estimates rather than precise subject-specific localizations.

### 2.4. fNIRS Data Analysis

fNIRS data preprocessing and analysis were conducted in MATLAB R2018a (The MathWorks, Natick, MA, USA), primarily using the HOMER2 and NIRS-KIT toolboxes. First, channel quality control was performed using the enPruneChannels function in HOMER2, with the signal-to-noise ratio (SNR) threshold set to 5. If more than 50% of all channels were identified as invalid for a given participant, that participant’s data were excluded from subsequent analyses. The raw light-intensity signals were then converted to optical density (OD) signals using the hmrIntensity2OD function.

Motion artifacts were first detected using the hmrMotionArtifactByChannel function, with the parameters set to tMotion = 1 s, tMask = 1 s, STDEVthresh = 20, and AMPthresh = 5. For segments identified as containing motion artifacts, spline interpolation (MotionCorrectSpline) was applied at the OD level (Zhao et al., 2023). The OD signals were then band-pass filtered at 0.01–0.10 Hz to attenuate low-frequency baseline drift and higher-frequency physiological noise, including cardiac and respiratory fluctuations (Carius et al., 2023).

The filtered OD signals were converted into changes in oxyhemoglobin (HbO) and deoxyhemoglobin (HbR) concentrations according to the modified Beer–Lambert law (MBLL). Differential pathlength factors (DPFs) were calculated separately for each participant and for each of the two near-infrared wavelengths used by the device, using the age- and wavelength-dependent formulation proposed by Scholkmann and Wolf (2013). These individualized DPF values were applied during the conversion from OD signals to HbO/HbR concentration changes to reduce potential biases in optical pathlength estimation arising from age- and wavelength-related differences. After obtaining the HbO and HbR signals, correlation-based signal improvement (CBSI) was further applied to correct residual motion artifacts.

Following preprocessing, the hemoglobin concentration time series were imported into NIRS-KIT. A general linear model (GLM) was constructed for each experimental condition using regressors time-locked to video onset with a duration of 3 s, corresponding to the 3000 ms video observation period; the subsequent scoring-response period was not included. The task regressors were generated by convolving the stimulus functions with the canonical hemodynamic response function implemented in SPM, modeled as a combination of two gamma functions. The GLM yielded condition-specific regression coefficients (β values) for each channel. For group-level statistical analyses, HbO β values were extracted for each participant, channel, and experimental condition. Thus, cortical activation was not quantified using raw HbO concentration changes or mean HbO values within a predefined time window. These β values primarily reflected scoring-related action observation and task-specific information processing during the video observation period rather than activity specifically associated with the subsequent score-entry period. Given that HbO generally exhibits a higher signal-to-noise ratio and greater sensitivity to task-related hemodynamic changes, HbO β values were used as the primary index of task-related cortical activation.

### 2.5. Statistical Analysis

For the behavioral data, accuracy (ACC) and reaction time (RT) were analyzed separately using 2 (judge group: expert vs. less-experienced) × 2 (decision type: execution scoring vs. difficulty scoring) mixed-design analyses of variance (ANOVAs). Judge group was treated as the between-subjects factor, whereas decision type was treated as the within-subjects factor. Effect sizes for the omnibus effects were reported as partial eta squared (ηp^2^). To further examine between-group differences under each decision type, four planned simple-effects comparisons were conducted: between-group comparisons of ACC and RT separately within the execution and difficulty scoring conditions. To control the family-wise error rate across these four planned comparisons, the Holm adjustment was applied to the corresponding p values. Standardized between-group effect sizes were expressed as Hedges’ g, together with 95% confidence intervals. As a sensitivity analysis addressing the different binary correctness criteria used for the two scoring tasks, mean absolute scoring error was calculated as the absolute difference between each participant’s response and the corresponding expert reference score on each trial and then averaged separately for the execution and difficulty scoring conditions. Mean absolute error was analyzed using the same 2 (judge group) × 2 (decision type) mixed-design ANOVA. Following a significant judge group × decision type interaction, between-group simple-effects comparisons were conducted separately within the execution and difficulty scoring conditions. The Holm adjustment was applied across these two follow-up comparisons, and Hedges’ g with 95% confidence intervals was reported. The final behavioral analyses included 23 expert judges and 26 less-experienced judges. Behavioral statistical analyses were performed using IBM SPSS Statistics 26.0.

For the fNIRS data, HbO β values estimated from the individual-level general linear model (GLM) were used as the primary index of task-related cortical activation. For each channel, HbO β values were analyzed using a 2 (judge group: expert vs. less-experienced) × 2 (decision type: execution scoring vs. difficulty scoring) mixed-design ANOVA. The main effects of judge group and decision type, as well as the judge group × decision type interaction, were tested, with ηp^2^ reported as the effect size measure. To control for false positives arising from channel-wise multiple comparisons, false discovery rate (FDR) correction was applied separately to the p values for each of the three effects across all channels, with an FDR-corrected *p* < .05 considered statistically significant. For channels that remained significant after FDR correction, simple-effects analyses were further conducted to examine differences in HbO β values between the execution scoring and difficulty scoring conditions within each judge group.

To further explore the relationship between scoring performance and task-related brain activity, fNIRS channels that remained significant after multiple-comparison correction were defined as channels of interest. Within the expert and less-experienced groups separately, Pearson correlation analyses were conducted to examine the associations between HbO β values and behavioral measures (ACC and RT). Fisher’s r-to-z tests were then used to compare the correlation coefficients between the two groups. All statistical tests were two-tailed, with the significance level set at *p* < .05.

## 3. Results

### 3.1. Behavioral Performance

**Figure 3:**
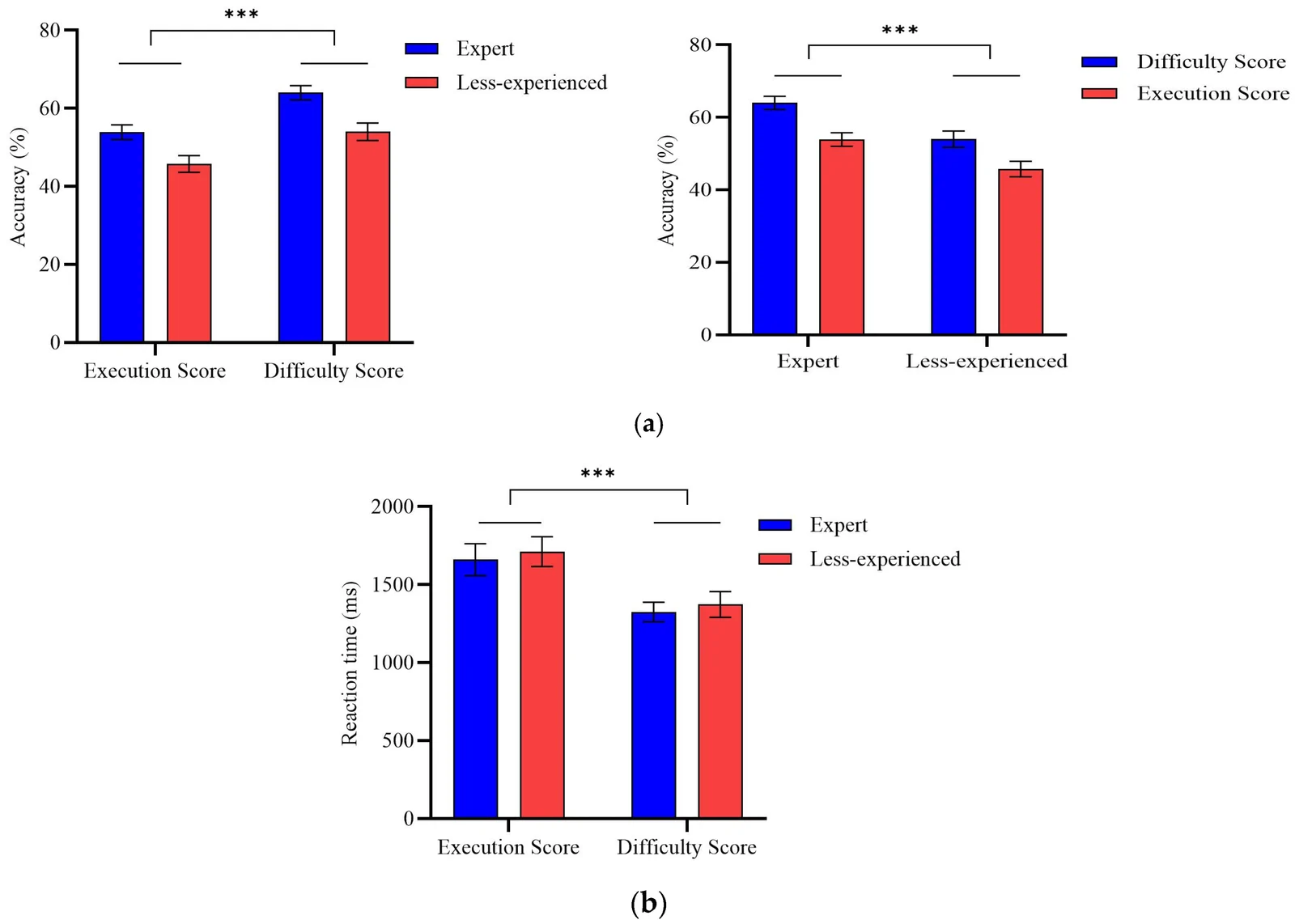
Behavioral performance across judge groups and decision types. (**a**) Mean accuracy for expert and less-experienced judges during execution score and difficulty score decisions, displayed by decision type and by judge group. (**b**) Mean reaction time for expert and less-experienced judges during execution score and difficulty score decisions. Error bars represent standard errors. Asterisks indicate significant main effects from the mixed-design ANOVA: *** *p* < .001.

For accuracy, the 2 × 2 mixed-design ANOVA revealed a significant main effect of judge group, *F* (1, 47) = 13.63, *p* < .001, ηp^2^ = 0.23, indicating that expert judges showed higher overall accuracy than less-experienced judges. The main effect of decision type was also significant, *F* (1, 47) = 32.59, *p* < .001, ηp^2^ = 0.41, indicating a significant difference in overall accuracy between execution score and difficulty score decisions. The judge group × decision type interaction was not significant, *F* (1, 47) = 0.31, *p* = .580, ηp^2^ = 0.007 (Table 2 and Table 3; Figure 3a).

Planned simple-effects analyses further showed that, in the execution scoring condition, expert judges had significantly higher accuracy than less-experienced judges, with a mean difference of 8.14 percentage points, 95% CI [2.25, 14.03], *t* (47) = 2.78, *p* = .008, Holm-adjusted *p* = .024, Hedges’ g = 0.78, 95% CI [0.21, 1.36]. In the difficulty scoring condition, expert judges likewise showed significantly higher accuracy than less-experienced judges, with a mean difference of 10.01 percentage points, 95% CI [3.94, 16.08], *t* (47) = 3.32, *p* = .002, Holm-adjusted *p* = .008, Hedges’ g = 0.93, 95% CI [0.35, 1.52]. Thus, although the judge group × decision type interaction was not significant, expert judges demonstrated higher scoring accuracy in both scoring tasks (Table 4).

For reaction time, the main effect of judge group was not significant, *F* (1, 47) = 0.28, *p* = .598, ηp^2^ = 0.006, whereas the main effect of decision type was significant, *F* (1, 47) = 15.82, *p* < .001, ηp^2^ = 0.25. The judge group × decision type interaction was also not significant, *F* (1, 47) < 0.01, *p* = .989, ηp^2^ < 0.001 (Table 2 and Table 3; Figure 3b). Planned simple-effects analyses showed no significant between-group difference in reaction time for execution scoring, with a mean difference of −51.23 ms, 95% CI [−340.50, 238.04], *t* (47) = −0.36, *p* = .723, Holm-adjusted *p* = 1.000, Hedges’ g = −0.10, 95% CI [−0.65, 0.45]. Similarly, no significant between-group difference was observed for difficulty scoring, with a mean difference of −48.88 ms, 95% CI [−264.52, 166.76], *t* (47) = −0.46, *p* = .651, Holm-adjusted *p* = 1.000, Hedges’ g = −0.13, 95% CI [−0.68, 0.42]. Overall, professional experience was primarily reflected in differences in scoring accuracy rather than in between-group differences in reaction time (Table 4).

To examine whether the behavioral findings were robust to the different binary correctness criteria used for execution and difficulty scoring, an additional sensitivity analysis was conducted using mean absolute scoring error. Absolute error was defined as the absolute difference between each participant’s response and the corresponding expert reference score on each trial, thereby providing a common continuous metric across the two decision types. All trials were retained, including difficulty trials with a reference score of zero. The 2 × 2 mixed-design ANOVA revealed a significant main effect of judge group, *F* (1, 47) = 7.46, *p* = .009, ηp^2^ = 0.137, with expert judges showing lower absolute error overall than less-experienced judges. The main effect of decision type was also significant, *F* (1, 47) = 572.90, *p* < .001, ηp^2^ = 0.924, with lower absolute error for difficulty scoring than for execution scoring. In addition, the judge group × decision type interaction was significant, *F* (1, 47) = 4.11, *p* = .048, ηp^2^ = 0.080.

Follow-up comparisons indicated significantly lower absolute error in expert judges for both execution scoring (Holm-adjusted *p* = .028) and difficulty scoring (Holm-adjusted *p* = .033), with a larger between-group difference for execution scoring than for difficulty scoring (Supplementary Table S2).

### 3.2. fNIRS Results

#### 3.2.1. Expertise-Dependent Modulation of Cortical Activation by Decision Type

**Figure 4:**
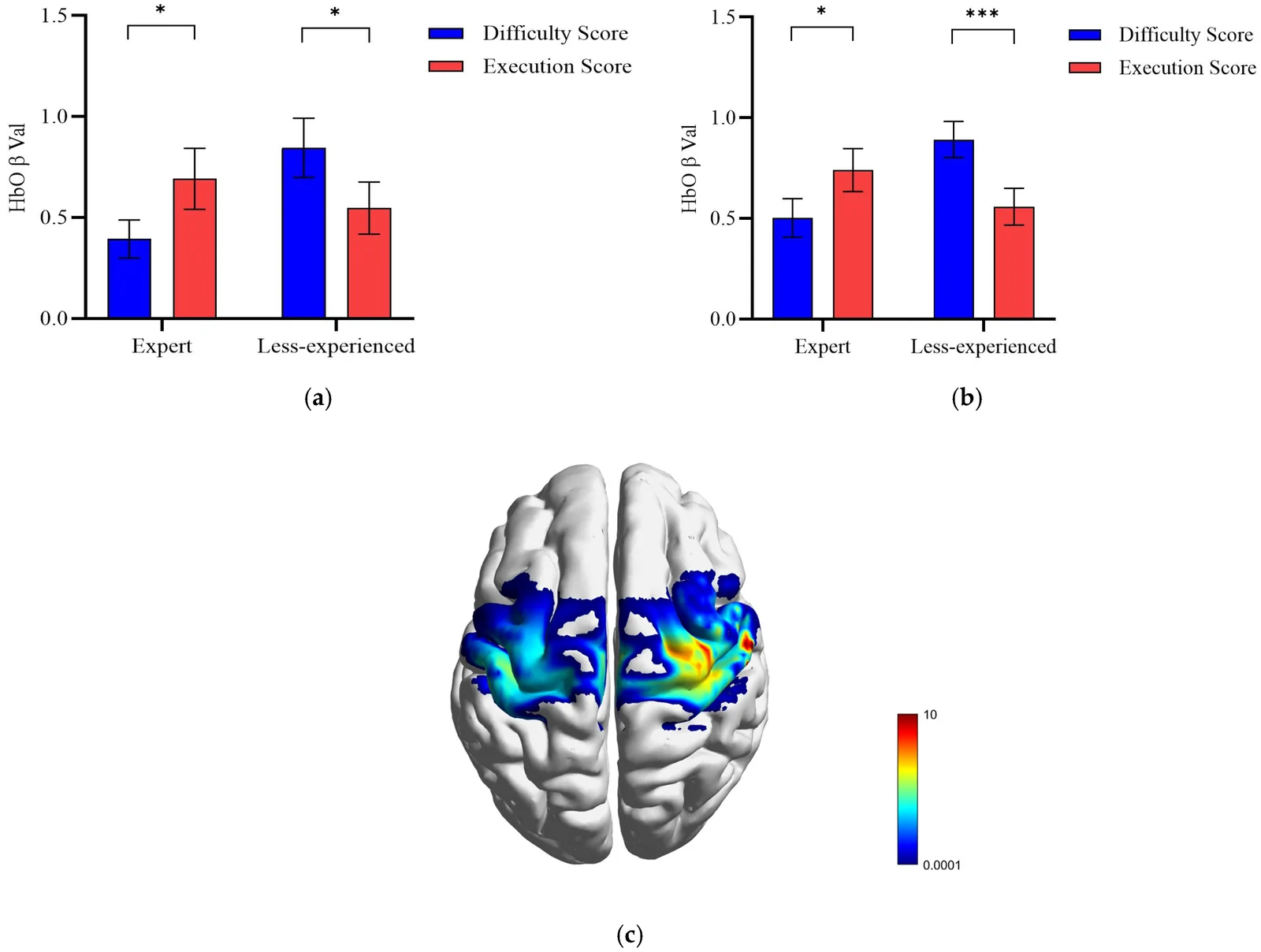
Judge group × decision type interaction effects on task-related HbO β values. (**a**) Mean HbO β values in CH13, primarily localized to the right precentral gyrus, for expert and less-experienced judges during the video observation period under the difficulty score and execution score conditions. (**b**) Mean HbO β values in CH19, primarily localized to the right postcentral gyrus, for expert and less-experienced judges during the video observation period under the difficulty score and execution score conditions. Error bars represent standard errors. Asterisks indicate significant within-group simple effects based on uncorrected *p* values between difficulty score and execution score conditions: * *p* < .05, *** *p* < .001. (**c**) Cortical F-value statistical parametric map for the judge group × decision type interaction. The map highlights channels showing significant interaction effects after channel-wise FDR correction, including CH13 and CH19.

The judge group × decision type interaction was primarily observed in CH13 and CH19. CH13 was mainly localized to the right precentral gyrus, whereas CH19 was mainly localized to the right postcentral gyrus (Figure 4). The mixed-design ANOVA revealed a significant judge group × decision type interaction in CH13, *F* (1, 47) = 9.43, *p* = .004, ηp^2^ = 0.167; this interaction remained significant after channel-wise FDR correction (FDR-adjusted *p* = .035). CH19 likewise showed a significant judge group × decision type interaction, *F* (1, 47) = 21.66, *p* < .001, ηp^2^ = 0.315, which also remained significant after FDR correction (FDR-adjusted *p* < .001). In contrast, neither the main effect of judge group nor the main effect of decision type reached significance in either channel.

Further simple-effects analyses showed a crossover pattern of task-related modulation in CH13. Expert judges exhibited significantly lower HbO β values during the video observation period under the difficulty score condition than during the video observation period under the execution score condition (difficulty: M ± SD = 0.394 ± 0.452; execution: 0.692 ± 0.723), with a mean difference of −0.298, 95% CI [−0.583, −0.014], *t* (47) = −2.11, *p* = .040. Less-experienced judges showed the opposite pattern, with significantly higher HbO β values during the video observation period under the difficulty score condition than during the video observation period under the execution score condition (difficulty: 0.845 ± 0.747; execution: 0.547 ± 0.658), with a mean difference of 0.297, 95% CI [0.030, 0.565], *t* (47) = 2.24, *p* = .030.

CH19 exhibited an even more pronounced crossover pattern. Expert judges showed significantly lower HbO β values during the video observation period under the difficulty score condition than during the video observation period under the execution score condition (difficulty: M ± SD = 0.503 ± 0.459; execution: 0.740 ± 0.511), with a mean difference of −0.238, 95% CI [−0.417, −0.058], *t* (47) = −2.66, *p* = .011, ηp^2^ = 0.130. In contrast, less-experienced judges showed higher HbO β values during the video observation period under the difficulty score condition than during the video observation period under the execution score condition (difficulty: 0.892 ± 0.456; execution: 0.558 ± 0.467), with a mean difference of 0.334, 95% CI [0.165, 0.503], *t* (47) = 3.97, *p*< .001, ηp^2^ = 0.251. Overall, CH13 and CH19 showed a consistent crossover pattern during scoring-related action observation: expert judges exhibited greater task-related cortical activation under the execution score requirement, whereas less-experienced judges exhibited greater activation under the difficulty score requirement. As an additional descriptive convergence check, CBSI-processed and pre-CBSI HbO/HbR time courses for CH13 and CH19 across both judge groups and scoring conditions are provided in Supplementary Figure S1, allowing visual comparison of the HbR traces before and after CBSI processing; no additional inferential conclusions were drawn from these traces.

#### 3.2.2. Associations Between Behavioral Performance and fNIRS Measures in Aerobic Gymnastics Judges with Different Expertise Levels

To further examine the relationship between behavioral performance and task-related brain activation, CH13 and CH19, which showed significant judge group × decision type interactions after FDR correction in the preceding analyses, were selected as channels of interest. Within the expert and less-experienced groups separately, Pearson correlations were calculated between HbO β values and behavioral measures (scoring accuracy and reaction time) under the corresponding decision conditions. Fisher’s r-to-z tests were then conducted to compare the correlation coefficients between the two groups.

Within-group Pearson correlation analyses showed that, in the less-experienced group, HbO β values in CH13 were significantly positively correlated with reaction time during the video observation period under the execution score condition (*r* = 0.634, *p* < .001) and with scoring accuracy during the video observation period under the difficulty score condition (*r* = 0.408, *p* = .039). In CH19, HbO β values during the video observation period under the execution score condition were also significantly positively correlated with reaction time (*r* = 0.457, *p* = .019). In the expert group, none of the correlations between HbO β values and the corresponding behavioral measures reached statistical significance (all *p*s > 0.05).

Fisher’s r-to-z tests showed that only the correlation between HbO β values and reaction time in CH19 during the video observation period under the execution score condition differed significantly between the two groups (*z* = −1.98, uncorrected *p* = .048). Specifically, the less-experienced group showed a positive correlation (*r* = 0.457, *p* = .019), whereas the correlation was weak in the expert group (*r* = −0.112, *p* = .612). None of the remaining between-group comparisons reached statistical significance. Given that this finding emerged from multiple exploratory tests and was not subjected to the corresponding multiple-comparison correction, it should be regarded as hypothesis-generating evidence for future research rather than as support for a stable between-group difference in brain–behavior coupling.

## 4. Discussion

This study showed an experience-related behavioral advantage and task-dependent differences in sensorimotor cortical recruitment during aerobic gymnastics scoring. The neural findings were characterized by judge group × decision type interactions rather than a uniform expertise-related increase or decrease in activation.

First, at the behavioral level, expert judges showed better scoring performance than less-experienced judges. Under the original task-specific binary accuracy criteria, difficulty score decisions showed higher accuracy than execution score decisions, whereas execution score decisions required longer reaction times. Because execution responses were considered correct within ±1 point of the expert reference whereas difficulty responses required an exact match, we conducted a sensitivity analysis using mean absolute scoring error as a common continuous metric. This analysis again showed an overall expert advantage, with significantly lower absolute error in expert judges in both scoring conditions, while also revealing a decision type effect and a judge group × decision type interaction, with a larger expertise-related difference during execution scoring. Cross-task differences should nevertheless be interpreted cautiously because the two scoring dimensions differ in their underlying score structures, including the presence of zero-reference trials in the difficulty condition. The longer reaction times for execution scoring may reflect its greater reliance on continuous evaluation of movement stability, fluency, and control quality, whereas difficulty scoring may depend more on matching action categories, technical structures, and rule-based criteria (Seger & Miller, 2010; Maddox et al., 2010; International Gymnastics Federation [FIG], 2024; Scully, 1986).

Second, the fNIRS results further indicated that the influence of professional experience on the neural mechanisms of judging was primarily reflected in the interaction between judge group and decision type, rather than in a simple overall increase or decrease in cortical activation. Importantly, the GLM modeled only the 3000 ms video observation period, not the subsequent score-entry period; therefore, the HbO β effects primarily reflect scoring-related action observation and task-specific processing under the cued scoring requirement rather than direct neural correlates of the post-video response. CH13 and CH19 were primarily localized to the right precentral gyrus and right postcentral gyrus, respectively, and both showed significant judge group × decision type interactions, suggesting that the modulatory effect of professional experience across the two scoring tasks involves multiple components of sensorimotor processing. The precentral gyrus has been implicated in motor representations, action prediction, and motor processing during action observation, whereas the postcentral gyrus contributes to action-related somatosensory processing and sensorimotor integration (Jeannerod, 2001; Grèzes & Decety, 2001; Rizzolatti & Craighero, 2004; Caspers et al., 2010; Stadler et al., 2011). Therefore, the concurrent interaction effects observed in CH13 and CH19 suggest that judges with different levels of professional experience may differ in how they recruit neural processes related to action representation and sensory-information integration during scoring-related action observation under the execution score and difficulty score requirements.

Among these findings, CH19, primarily localized to the right postcentral gyrus, exhibited a relatively clear crossover pattern of activation: expert judges showed greater task-related activation during the video observation period under the execution score condition than during the video observation period under the difficulty score condition, whereas less-experienced judges showed greater activation during the video observation period under the difficulty score condition. For less-experienced judges, difficulty score judgments may still require greater reliance on online analysis of movement structure, rule matching, and sensorimotor mapping (Bard et al., 1980; Ste-Marie, 1999, 2000; Pizzera, 2012), thereby increasing the recruitment of relevant sensorimotor resources. With the accumulation of professional experience, categorical knowledge of difficulty elements, rule structures, and action representations may become increasingly proceduralized, reducing experts’ reliance on online analysis (Olsson & Lundström, 2013; Guo et al., 2019; Li & Smith, 2021). In contrast, execution score judgments require continuous evaluation of movement quality, stability, and control-related information. The greater sensorimotor-related activation observed in experts under this condition may therefore reflect more refined and selective task-relevant processing of movement quality information (Yang, 2015; Karlinsky et al., 2017). Taken together, the expert advantage does not appear to be characterized by uniformly lower activation across tasks, but rather by task-dependent adjustment in the recruitment of relevant neural resources.

This crossover pattern suggests that experience-related differences in neural activity are task-dependent rather than reflecting a uniform neural efficiency effect. Consistent with this view, previous studies have shown that expertise-related activation differences may vary, or even reverse, across different task demands (Dunst et al., 2014; Calvo-Merino et al., 2005; Del Percio et al., 2008; Babiloni et al., 2010). Accordingly, the present findings do not support a classical neural efficiency account in which experts consistently exhibit lower activation across all tasks. Instead, they are more consistent with a task-dependent selective-recruitment or task–resource matching perspective.

The exploratory between-group difference in CH19 brain–behavior coupling should be interpreted cautiously because it was not corrected for multiple comparisons and requires replication in larger independent samples.

Several limitations should be acknowledged. The cross-sectional design and substantial age difference between groups preclude causal attribution of the observed effects to professional experience; individualized age-dependent DPF correction cannot remove possible age-related cognitive or hemodynamic differences. The behavioral criterion was not fully independent because the criterion judges and expert participants came from the same professional population, while official competition D- and E-scores could not provide clip-level reference values; therefore, greater expert–criterion agreement may partly reflect shared scoring conventions, and any resulting inflation could not be quantified. In addition, D-, E-, and other jury roles are assigned on a competition-specific basis rather than representing stable specializations; cumulative role-specific exposure was not quantified and may have contributed to individual differences in behavioral or cortical responses. The fNIRS montage provided limited coverage of broader decision-making networks and lacked short-separation channels, so systemic physiological influences cannot be excluded. Finally, the exploratory brain–behavior analyses require replication in larger, age-matched samples.

## 5. Conclusions

This study compared behavioral performance and sensorimotor-related cortical activity during the video observation period under difficulty score and execution score requirements in aerobic gymnastics judges with different levels of experience. The results showed that expert judges achieved higher scoring accuracy than less-experienced judges in both scoring tasks, and this expert advantage was also supported by the sensitivity analysis using a common absolute error metric; under the current accuracy criteria, the two tasks also differed in accuracy and reaction time. The neural results revealed crossover interactions between judge group and decision type in the right precentral and postcentral gyri, suggesting that the two groups may recruit sensorimotor resources differently in response to distinct task demands. Given the age differences between the groups, the cross-sectional design, and the limitations of the scoring measures, these findings should be interpreted as experience-related between-group differences rather than direct evidence of a causal effect of long-term professional experience.

## Figures and Tables

**Table 1:** Regions of interest (ROIs) and primary anatomical localization of the 20 fNIRS channels based on the AAL atlas.

Regions of Interest (ROIs)		Hemisphere	Channel	Maximum Overlap (%)
1	Frontal superior gyrus (SFG)	L	CH1	45.70%
		R	CH11	81.32%
2	Middle frontal gyrus (MFG)	L	CH4	100.00%
		R	CH14	98.73%
3	Precentral gyrus (PreCG)	L	CH2	61.84%
		L	CH3	78.37%
		L	CH5	86.87%
		R	CH12	85.07%
		R	CH13	63.10%
		R	CH15	65.06%
4	Inferior frontal gyrus (IFG)	L	CH6	39.79%
		R	CH16	58.10%
5	Postcentral gyrus (PoCG)	L	CH7	67.84%
		L	CH8	41.60%
		L	CH9	93.31%
		L	CH10	53.82%
		R	CH17	73.08%
		R	CH18	60.23%
		R	CH19	86.82%
		R	CH20	36.77%

**Table 2:** Accuracy and reaction time of expert and less-experienced aerobic gymnastics judges in execution score and difficulty score decisions.

	Judge Group	Execution Score (Mean ± SE, 95% CI)	Difficulty Score (Mean ± SE, 95% CI)
ACC(%)	Expert judges	53.91 ± 1.90 [49.97–57.85]	64.39 ± 1.86 [60.53–68.25]
	Less-experienced judges	45.77 ± 2.18 [41.28–50.26]	54.38 ± 2.31 [49.63–59.14]
RT(ms)	Expert judges	1660.30 ± 104.95 [1442.64–1877.95]	1324.57 ± 63.87 [1192.10–1457.03]
	Less-experienced judges	1711.53 ± 98.34 [1508.99–1914.07]	1373.45 ± 83.45 [1201.59–1545.31]

Notes: ACC = accuracy; RT = reaction time; CI = confidence intervals; expert judges, *n* = 23; less-experienced judges, *n* = 26.

**Table 3:** Analysis of variance of accuracy and reaction time in execution score and difficulty score decisions among expert and less-experienced aerobic gymnastics judges.

	ACC				RT			
	F	Df	*p*	η^2^_p_	F	Df	*p*	η^2^_p_
Judge group	13.63	1.47	<0.001	0.23	0.28	1.47	0.598	0.006
Decision type	32.59	1.47	<0.001	0.41	15.82	1.47	<0.001	0.25
Judge group × Decision type	0.31	1.47	0.580	0.007	0.00	1.47	0.989	0.00

**Table 4:** Simple-effects comparisons between expert and less-experienced judges for accuracy and reaction time in execution score and difficulty score decisions.

	Decision Type	Mean Difference	95% CI	*t* (47)	Raw *p*	Holm- Adjusted *p*	Hedges’ g	95% CI for g
ACC (%)	Execution score	8.14	[2.25, 14.03]	2.78	.008	.024	0.78	[0.21, 1.36]
ACC (%)	Difficulty score	10.01	[3.94, 16.08]	3.32	.002	.008	0.93	[0.35, 1.52]
RT (ms)	Execution score	−51.23	[−340.50, 238.04]	−0.36	.723	1.000	−0.10	[−0.65, 0.45]
RT (ms)	Difficulty score	−48.88	[−264.52, 166.76]	−0.46	.651	1.000	−0.13	[−0.68, 0.42]

Note: Positive ACC values indicate higher accuracy for expert judges than for less-experienced judges; negative RT values indicate shorter reaction times for expert judges. Hedges’ g is reported as the standardized effect size with 95% confidence intervals.

## Data Availability

The data presented in this study are not publicly available due to ethical and confidentiality restrictions. The dataset includes sensitive information related to official performance evaluations of aerobic gymnastics judges conducted within an institutional talent development program. Anonymized data may be made available by the corresponding author upon reasonable request and with permission from the relevant governing body.

## Supplementary Materials

The following supporting information can be downloaded at: https://www.mdpi.com/article/10.3390/jintelligence14100245/s1. Figure S1: CBSI-processed and pre-CBSI HbO/HbR time courses for CH13 and CH19 across judge groups and scoring conditions. Table S1: Channel-wise mixed-design ANOVA results for HbO β values with raw and Benjamini–Hochberg FDR-adjusted *p* values. Table S2: Sensitivity analysis of scoring performance using mean absolute error.

## References

[B1-jintelligence-14-00245] Babiloni C., Marzano N., Infarinato F., Iacoboni M., Rizza G., Aschieri P., Cibelli G., Soricelli A., Eusebi F., Del Percio C. (2010). “Neural efficiency” of experts’ brain during judgment of actions: A high-resolution EEG study in elite and amateur karate athletes. Behavioural Brain Research.

[B2-jintelligence-14-00245] Bard C., Fleury M., Carrière L., Hallé M. (1980). Analysis of gymnastics judges’ visual search. Research Quarterly for Exercise and Sport.

[B3-jintelligence-14-00245] Calvo-Merino B., Glaser D. E., Grèzes J., Passingham R. E., Haggard P. (2005). Action observation and acquired motor skills: An fMRI study with expert dancers. Cerebral Cortex.

[B4-jintelligence-14-00245] Carius D., Herold F., Clauß M., Kaminski E., Wagemann F., Sterl C., Ragert P. (2023). Increased cortical activity in novices compared to experts during table tennis: A whole-brain fNIRS study using threshold-free cluster enhancement analysis. Brain Topography.

[B5-jintelligence-14-00245] Caspers S., Zilles K., Laird A. R., Eickhoff S. B. (2010). ALE meta-analysis of action observation and imitation in the human brain. NeuroImage.

[B6-jintelligence-14-00245] Catteeuw P., Helsen W., Gilis B., Wagemans J. (2009). Decision-making skills, role specificity, and deliberate practice in association football refereeing. Journal of Sports Sciences.

[B7-jintelligence-14-00245] Chase W. G., Simon H. A. (1973). Perception in chess. Cognitive Psychology.

[B8-jintelligence-14-00245] Del Percio C., Rossini P. M., Marzano N., Iacoboni M., Infarinato F., Aschieri P., Lino A., Fiore A., Toran G., Babiloni C., Eusebi F. (2008). Is there a “neural efficiency” in athletes? A high-resolution EEG study. NeuroImage.

[B9-jintelligence-14-00245] Dunst B., Benedek M., Jauk E., Bergner S., Koschutnig K., Sommer M., Ischebeck A., Spinath B., Arendasy M., Bühner M., Freudenthaler H., Neubauer A. C. (2014). Neural efficiency as a function of task demands. Intelligence.

[B10-jintelligence-14-00245] Faul F., Erdfelder E., Lang A.-G., Buchner A. (2007). G*Power 3: A flexible statistical power analysis program for the social, behavioral, and biomedical sciences. Behavior Research Methods.

[B11-jintelligence-14-00245] Flessas K., Mylonas D., Panagiotaropoulou G., Tsopani D., Korda A., Siettos C., Di Cagno A., Evdokimidis I., Smyrnis N. (2015). Judging the judges’ performance in rhythmic gymnastics. Medicine & Science in Sports & Exercise.

[B12-jintelligence-14-00245] Gobet F. (1998). Expert memory: A comparison of four theories. Cognition.

[B13-jintelligence-14-00245] Grèzes J., Decety J. (2001). Functional anatomy of execution, mental simulation, observation, and verb generation of actions: A meta-analysis. Human Brain Mapping.

[B14-jintelligence-14-00245] Guo Z., Li A., Yu L. (2019). Neural efficiency and acquired motor skills: An fMRI study of expert athletes. Frontiers in Psychology.

[B15-jintelligence-14-00245] Herold F., Wiegel P., Scholkmann F., Müller N. (2018). Applications of functional near-infrared spectroscopy (fNIRS) neuroimaging in exercise–cognition science: A systematic, methodology-focused review. Journal of Clinical Medicine.

[B16-jintelligence-14-00245] International Gymnastics Federation [FIG] (2024). Aerobic gymnastics code of points 2025–2028.

[B17-jintelligence-14-00245] Jeannerod M. (2001). Neural simulation of action: A unifying mechanism for motor cognition. NeuroImage.

[B18-jintelligence-14-00245] Karlinsky A., Zentgraf K., Hodges N. J. (2017). Action-skilled observation: Issues for the study of sport expertise and the brain. Progress in Brain Research.

[B19-jintelligence-14-00245] Kittel A., Larkin P., Elsworthy N., Spittle M. (2019). Video-based testing in sporting officials: A systematic review. Psychology of Sport and Exercise.

[B20-jintelligence-14-00245] Klein G. (2008). Naturalistic decision making. Human Factors: The Journal of the Human Factors and Ergonomics Society.

[B21-jintelligence-14-00245] Klein G. (2015). A naturalistic decision making perspective on studying intuitive decision making. Journal of Applied Research in Memory and Cognition.

[B22-jintelligence-14-00245] Lee J., Knowles Z., Whitehead A. E. (2019). Exploring the use of think aloud within Women’s artistic gymnastics judging education. Psychology of Sport and Exercise.

[B23-jintelligence-14-00245] Li L., Smith D. M. (2021). Neural efficiency in athletes: A systematic review. Frontiers in Behavioral Neuroscience.

[B24-jintelligence-14-00245] Maddox W. T., Pacheco J., Reeves M., Zhu B., Schnyer D. M. (2010). Rule-based and information-integration category learning in normal aging. Neuropsychologia.

[B25-jintelligence-14-00245] Mann D. T. Y., Williams A. M., Ward P., Janelle C. M. (2007). Perceptual-cognitive expertise in sport: A meta-analysis. Journal of Sport & Exercise Psychology.

[B26-jintelligence-14-00245] Milton F., Pothos E. M. (2011). Category structure and the two learning systems of COVIS: The two systems of COVIS. European Journal of Neuroscience.

[B27-jintelligence-14-00245] Moore L. J., Harris D. J., Sharpe B. T., Vine S. J., Wilson M. R. (2019). Perceptual-cognitive expertise when refereeing the scrum in rugby union. Journal of Sports Sciences.

[B28-jintelligence-14-00245] Neubauer A. C., Fink A. (2009). Intelligence and neural efficiency. Neuroscience & Biobehavioral Reviews.

[B29-jintelligence-14-00245] Neumann N., Lotze M., Eickhoff S. B. (2016). Cognitive expertise: An ALE meta-analysis. Human Brain Mapping.

[B30-jintelligence-14-00245] Oldfield R. C. (1971). The assessment and analysis of handedness: The Edinburgh inventory. Neuropsychologia.

[B31-jintelligence-14-00245] Olsson C.-J., Lundström P. (2013). Using action observation to study superior motor performance: A pilot fMRI study. Frontiers in Human Neuroscience.

[B32-jintelligence-14-00245] Pizzera A. (2012). Gymnastic judges benefit from their own motor experience as gymnasts. Research Quarterly for Exercise and Sport.

[B33-jintelligence-14-00245] Pizzera A. (2015). The role of embodied cognition in sports officiating. Movement & Sport Sciences–Science & Motricité.

[B34-jintelligence-14-00245] Pizzera A., Raab M. (2012). Perceptual judgments of sports officials are influenced by their motor and visual experience. Journal of Applied Sport Psychology.

[B35-jintelligence-14-00245] Plessner H., Haar T. (2006). Sports performance judgments from a social cognitive perspective. Psychology of Sport and Exercise.

[B36-jintelligence-14-00245] Raab M., Avugos S., Bar-Eli M., MacMahon C. (2021). The referee’s challenge: A threshold process model for decision making in sport games. International Review of Sport and Exercise Psychology.

[B37-jintelligence-14-00245] Rizzolatti G., Craighero L. (2004). The mirror-neuron system. Annual Review of Neuroscience.

[B38-jintelligence-14-00245] Russell S., Renshaw I., Davids K. (2019). How interacting constraints shape emergent decision-making of national-level football referees. Qualitative Research in Sport, Exercise and Health.

[B39-jintelligence-14-00245] Samuel R. D., Filho E., Galily Y. (2024). Attention allocation in elite football refereeing: Conceptual, empirical, and applied considerations. Journal of Cognitive Psychology.

[B40-jintelligence-14-00245] Scholkmann F., Wolf M. (2013). General equation for the differential pathlength factor of the frontal human head depending on wavelength and age. Journal of Biomedical Optics.

[B41-jintelligence-14-00245] Scully D. M. (1986). Visual perception of technical execution and aesthetic quality in biological motion. Human Movement Science.

[B42-jintelligence-14-00245] Seger C. A., Miller E. K. (2010). Category learning in the brain. Annual Review of Neuroscience.

[B43-jintelligence-14-00245] Shiffrin R. M., Schneider W. (1977). Controlled and automatic human information processing: II. Perceptual learning, automatic attending and a general theory. Psychological Review.

[B44-jintelligence-14-00245] Shui Y., Che X., Zhang Y., Ma N., Li J., You X., Wan B. (2024). The role of offensive processes and age development for female soccer players’ anticipation. Scientific Reports.

[B45-jintelligence-14-00245] Spitz J., Put K., Wagemans J., Williams A. M., Helsen W. F. (2016). Visual search behaviors of association football referees during assessment of foul play situations. Cognitive Research: Principles and Implications.

[B46-jintelligence-14-00245] Stadler W., Schubotz R. I., Von Cramon D. Y., Springer A., Graf M., Prinz W. (2011). Predicting and memorizing observed action: Differential premotor cortex involvement. Human Brain Mapping.

[B47-jintelligence-14-00245] Ste-Marie D. M. (1999). Expert-novice differences in gymnastic judging: An information-processing perspective. Applied Cognitive Psychology.

[B48-jintelligence-14-00245] Ste-Marie D. M. (2000). Expertise in women’s gymnastic judging: An observational approach. Perceptual and Motor Skills.

[B49-jintelligence-14-00245] Wu Y., Yang Z., Wang R., Zeng H., Zhang Q. (2024). A comparison of perceptual-cognitive skills in expert and non-expert sports officials: A systematic review and meta-analysis. Frontiers in Psychology.

[B50-jintelligence-14-00245] Yang J. (2015). The influence of motor expertise on the brain activity of motor task performance: A meta-analysis of functional magnetic resonance imaging studies. Cognitive, Affective, & Behavioral Neuroscience.

[B51-jintelligence-14-00245] Yücel M. A., Lühmann A. V., Scholkmann F., Gervain J., Dan I., Ayaz H., Boas D., Cooper R. J., Culver J., Elwell C. E., Eggebrecht A., Franceschini M. A., Grova C., Homae F., Lesage F., Obrig H., Tachtsidis I., Tak S., Tong Y., Wolf M. (2021). Best practices for fNIRS publications. Neurophotonics.

[B52-jintelligence-14-00245] Zentgraf K., Munzert J., Bischoff M., Newman-Norlund R. D. (2014). Simulation during observation of human actions—Theories, empirical studies, applications. Vision Research.

[B53-jintelligence-14-00245] Zhao Y., Luo H., Chen J., Loureiro R., Yang S., Zhao H. (2023). Learning based motion artifacts processing in fNIRS: A mini review. Frontiers in Neuroscience.

