# Supplementary material for: Professional Experience Moderates Behavioral Performance and Sensorimotor-Related Cortical Activity During Scoring Decision-Making in Aerobic Gymnastics Judges: An fNIRS Study
Source: J Intell. 2026 Oct 4;14(10):245. doi: 10.3390/jintelligence14100245 (PMC13705886; doi:10.3390/jintelligence14100245)
Supplement: Supplementary file 1 [file jintelligence-14-00245-s001.zip › jintelligence-4435513-supplementary.pdf]

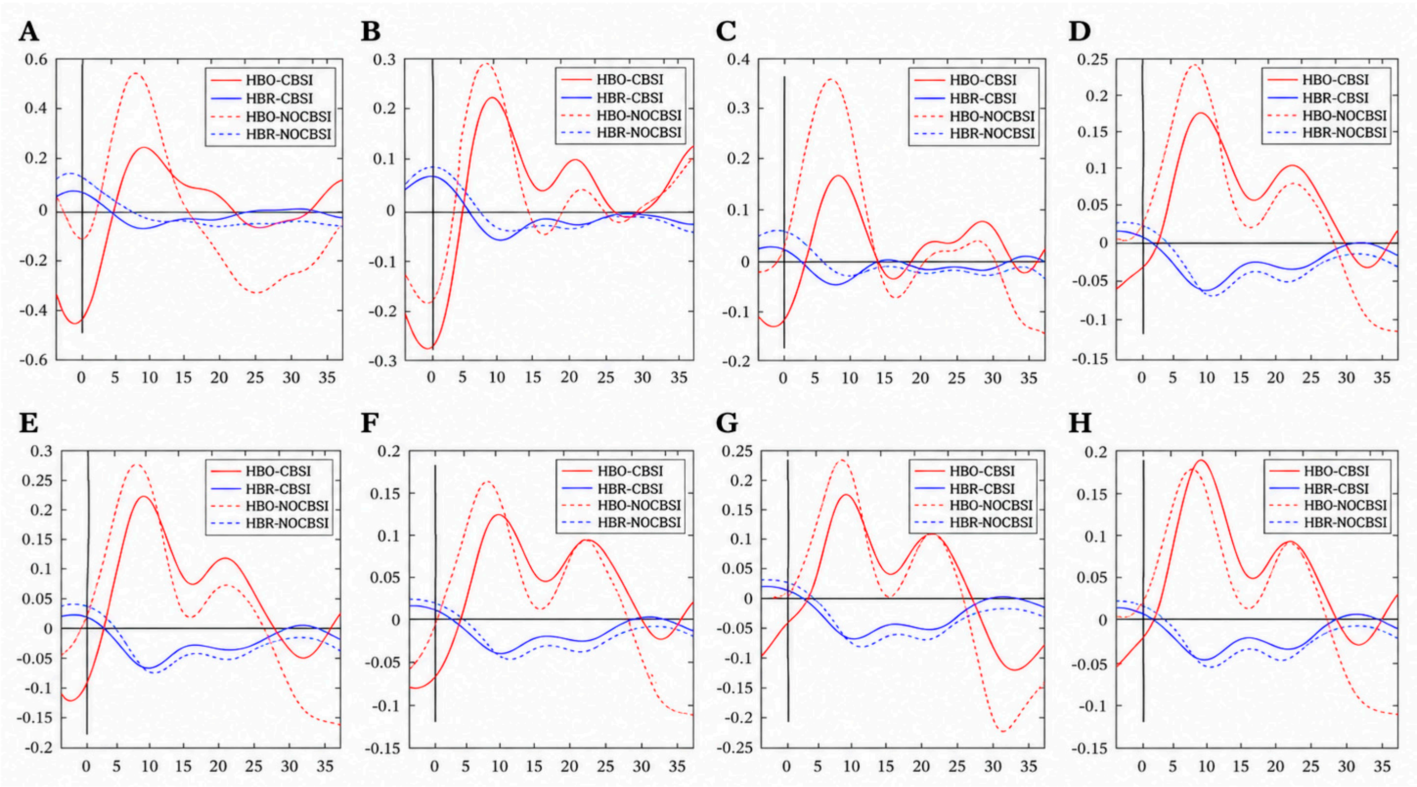

Supplementary Figure S1. CBSI-processed and pre-CBSI HbO/HbR time courses for CH13 and CH19 across judge groups and scoring conditions. From left to right and top to bottom, panels show: (A) CH13–expert–difficulty, (B) CH13–expert–execution, (C) CH13–less-experienced–difficulty, (D) CH13–less-experienced–execution, (E) CH19–expert–difficulty, (F) CH19–expert–execution, (G) CH19–less-experienced–difficulty, and (H) CH19–less-experienced–execution. Solid lines denote CBSI-processed signals and dashed lines denote signals before CBSI processing; red indicates HbO and blue indicates HbR. The vertical black line marks stimulus onset (0 s). The figure is provided as a descriptive convergence check and was not used for inferential statistical testing.

Supplement Table S1. Channel-wise mixed-design ANOVA results for HbO β values with raw and Benjamini–Hochberg FDR-adjusted p values.

	Main Effect of Judge Group				Main Effect of Decision Type				Group × Decision Type Interaction			
	F(1, 47)	p	FDR-adjusted p	ηp²	*F*(1, 47)	p	FDR-adjusted p	ηp²	*F*(1, 47)	p	FDR-adjusted p	ηp²
CH1	0.517	0.476	0.910	0.011	0.126	0.725	0.996	0.003	1.085	0.303	0.442	0.023
CH2	0.01	0.922	0.930	0	0.394	0.533	0.996	0.008	3.178	0.081	0.160	0.063
CH3	0.396	0.532	0.910	0.008	0.182	0.672	0.996	0.004	3.918	0.054	0.134	0.077
CH4	2.945	0.093	0.910	0.059	0.001	0.977	0.996	0	0.169	0.682	0.773	0.004
CH5	0.157	0.694	0.910	0.003	0.034	0.855	0.996	0.001	0.669	0.418	0.522	0.014
CH6	0.225	0.638	0.910	0.005	0.097	0.757	0.996	0.002	0.004	0.949	0.949	0
CH7	0.053	0.819	0.910	0.001	0.212	0.647	0.996	0.004	3.038	0.088	0.160	0.061
CH8	0.2	0.657	0.910	0.004	0.078	0.782	0.996	0.002	6.274	0.016	0.101	0.118
CH9	0.135	0.715	0.910	0.003	1.32	0.256	0.996	0.027	0.007	0.933	0.949	0
CH10	0.545	0.464	0.910	0.011	2.1	0.154	0.996	0.043	0.155	0.695	0.773	0.003
CH11	0.053	0.819	0.910	0.001	0	0.994	0.996	0	1.057	0.309	0.442	0.022
CH12	0.008	0.93	0.930	0	0.018	0.893	0.996	0	5.111	0.028	0.114	0.098
CH13	0.898	0.348	0.910	0.019	0	0.996	0.996	0	9.431	**0.004**	**0.035**	0.167
CH14	0.86	0.359	0.910	0.018	0.325	0.571	0.996	0.007	4.346	0.043	0.122	0.085
CH15	0.214	0.646	0.910	0.005	1.599	0.212	0.996	0.033	4.52	0.039	0.122	0.088
CH16	1.819	0.184	0.910	0.037	0.009	0.924	0.996	0	0.708	0.404	0.522	0.015
CH17	3.621	0.063	0.910	0.072	1.449	0.235	0.996	0.03	3.351	0.074	0.160	0.067
CH18	0.098	0.756	0.910	0.002	1.1	0.3	0.996	0.023	5.777	0.02	0.101	0.109
CH19	0.729	0.397	0.910	0.015	0.616	0.437	0.996	0.013	21.657	**< 0.001**	**< 0.001**	0.315
CH20	0.892	0.35	0.910	0.019	0.846	0.362	0.996	0.018	1.277	0.264	0.440	0.026

Note: Benjamini–Hochberg FDR correction was applied separately to each of the three ANOVA effects across the 20 channels (m = 20 per correction family). Bold values indicate

effects that remained significant after FDR correction (adjusted p < .05).

Supplementary Table S2. Sensitivity analysis of scoring performance using mean absolute error.

Analysis	Expert Judges	Less-Experienced Judges	Mean Difference (Expert − Less-Experienced)	95% CI	Test Statistic	Raw p	Holm-adjusted p	Hedges' g (95% CI)
Execution absolute error	1.667 ± 0.368	2.030 ± 0.587	−0.363	[−0.649, −0.077]	t(47) = −2.55	.014	.028	−0.72 [−1.29, −0.15]
Difficulty absolute error	0.307 ± 0.135	0.418 ± 0.206	−0.111	[−0.213, −0.010]	t(47) = −2.20	.033	.033	−0.62 [−1.19, −0.05]

Omnibus mixed-design ANOVA

Effect	F(1, 47)	p	ηp^2
Judge group	7.46	.009	.137
Decision type	572.90	< .001	.924
Judge group × Decision type	4.11	.048	.080

Note：Mean absolute error was calculated as the absolute difference between each participant's score and the corresponding expert reference score on each trial and was averaged separately for the execution- and difficulty-scoring conditions. Lower values indicate greater agreement with the expert reference score. All trials were retained, including difficulty trials with a reference score of zero. Follow-up between-group simple-effects comparisons were conducted after the significant judge group × decision type interaction. Holm adjustment was applied across the two follow-up comparisons.
